# Practical tool to identify Spasticity-Plus Syndrome amongst patients with multiple sclerosis. Algorithm development based on a conjoint analysis

**DOI:** 10.3389/fneur.2024.1371644

**Published:** 2024-04-19

**Authors:** Óscar Fernández Fernández, Lucienne Costa-Frossard, Maria Luisa Martínez Ginés, Paloma Montero Escribano, José María Prieto González, Lluís Ramió-Torrentà, Yolanda Aladro, Ana Alonso Torres, Elena Álvarez Rodríguez, Andrés Labiano-Fontcuberta, Lamberto Landete Pascual, Ambrosio Miralles Martínez, Ester Moral Torres, Pedro Oliva-Nacarino

**Affiliations:** ^1^Department of Pharmacology, Faculty of Medicine, Institute of Biomedical Research of Malaga (IBIMA), University of Malaga, Málaga, Spain; ^2^Department of Neurology, Ramón y Cajal University Hospital, Ramón y Cajal Health Research Institute (IRYCIS), Universidad de Alcalá, Madrid, Spain; ^3^Department of Neurology, Gregorio Marañón General University Hospital, Madrid, Spain; ^4^Neuroimmunology and Multiple Sclerosis Unit, Department of Neurology, San Carlos Clinical University Hospital, Madrid, Spain; ^5^Department of Neurology, Clinical University Hospital of Santiago, Health Research Institute of Santiago de Compostela, Santiago de Compostela, Spain; ^6^Girona Neuroimmunology and Multiple Sclerosis Unit, Department of Neurology, Dr. Josep Trueta University Hospital and Santa Caterina Hospital, Girona-Salt, Spain; ^7^Neurodegeneration and Neuroinflammation Research Group, IDIBGI, Girona-Salt, Spain; ^8^Department of Medical Sciences, Faculty of Medicine, University of Girona, Girona, Spain; ^9^University Hospital of Getafe, European University of Madrid, University Hospital La Paz Research Institute (IdiPAZ), Madrid, Spain; ^10^Department of Neurology, Regional University Hospital of Malaga, Málaga, Spain; ^11^Department of Neurology, Álvaro Cunqueiro Hospital, Vigo, Spain; ^12^Department of Neurology, University Hospital 12 de Octubre, Madrid, Spain; ^13^Neurology Department, Dr. Peset University Hospital, Valencia, Spain; ^14^Department of Neurology, Infanta Sofia University Hospital, San Sebastián de los Reyes, Spain; ^15^Department of Neurology, Moises Broggi University Hospital, Barcelona, Spain; ^16^Department of Neurology, Central University Hospital of Asturias (HUCA), Institute of Health Research of the Principality of Asturias (ISPA), Oviedo, Spain

**Keywords:** multiple sclerosis, spasticity, Spasticity-Plus Syndrome, conjoint analysis, nabiximols, bladder dysfunction

## Abstract

**Introduction:**

The Spasticity-Plus Syndrome (SPS) in multiple sclerosis (MS) refers to a combination of spasticity and other signs/symptoms such as spasms, cramps, bladder dysfunction, tremor, sleep disorder, pain, and fatigue. The main purpose is to develop a user-friendly tool that could help neurologists to detect SPS in MS patients as soon as possible.

**Methods:**

A survey research based on a conjoint analysis approach was used. An orthogonal factorial design was employed to form 12 patient profiles combining, at random, the eight principal SPS signs/symptoms. Expert neurologists evaluated in a survey and a logistic regression model determined the weight of each SPS sign/symptom, classifying profiles as SPS or not.

**Results:**

72 neurologists participated in the survey answering the conjoint exercise. Logistic regression results of the survey showed the relative contribution of each sign/symptom to the classification as SPS. Spasticity was the most influential sign, followed by spasms, tremor, cramps, and bladder dysfunction. The goodness of fit of the model was appropriate (AUC = 0.816). Concordance between the experts’ evaluation vs. model estimation showed strong Pearson’s (*r* = 0.936) and Spearman’s (*r* = 0.893) correlation coefficients. The application of the algorithm provides with a probability of showing SPS and the following ranges are proposed to interpret the results: high (> 60%), moderate (30–60%), or low (< 30%) probability of SPS.

**Discussion:**

This study offers an algorithmic tool to help healthcare professionals to identify SPS in MS patients. The use of this tool could simplify the management of SPS, reducing side effects related with polypharmacotherapy.

## 1 Introduction

A syndrome in medicine is classically defined as a combination of signs and/or symptoms that forms a distinct clinical picture indicative of a particular disease or disorder (1). These signs and/or symptoms might typically be considered to have a common underlying pathophysiology, or respond to a specific therapy, despite variability in clinical presentations. The broader concept of ‘Spasticity-Plus Syndrome’ in multiple sclerosis (MS) has been recently developed and refers to the combination of spasticity with other symptoms such as spasms, cramps, bladder dysfunction, tremor, sleep disorder, pain and fatigue (2, 3). Identifying patients with MS who present several of these signs/symptoms simultaneously allows to treat them through a syndromic approach, which can provide a better risk–benefit than treating every symptom separately (2, 3).

Multiple sclerosis affects progressively different areas of the CNS and the musculoskeletal system, causing a wide range of symptoms which have a great impact on patients’ quality of life and patient independence, including challenges with daily activities, speech or swallowing difficulties, weakness, blurred vision, amongst others. However, traditional approaches to the research and clinical management of MS have focused mainly on disease-modifying therapies, with less attention being given to symptomatic therapies (4). In-depth symptom assessment is often performed in monographic MS visits rather than routine clinical visits (3).

From a clinical standpoint, targeting the co-occurrence of signs and symptoms of the SPS with a single therapy would simplify the management of symptoms, showing that, although they do not present the same pathophysiological mechanism, they do not necessarily require tailored therapeutic strategies (2). Specifically for spasticity-related MS, it has been observed in clinical trials and real-world studies that by using an oromucosal spray containing a mixture of 9-δ-tetrahydrocannabinol and cannabidiol (THC:CBD), nabiximols, which target CB1 and CB2 receptors to treat spasticity in MS patients, an control is gained over a broader range of symptoms that constitute the main proposal of the SPS (5–13). Unfortunately, certain signs and symptoms present unique pathophysiological mechanisms that require individual therapeutic strategies.

The major unmet needs in spasticity management are: (1) a unified framework for managing all the seemingly related functions/symptoms; (2) pharmacological treatments that can be used long term without losing efficacy or causing side effects; and (3) better pharmacological management of symptoms related to spasticity muscle tone, because the current management is fragmented and often requires polypharmacy, which can lead to side effects and drug interactions. Our previous work reflected the importance of a uniform and an active screening of symptoms, especially MS-related spasticity, which is often diagnosed too late (3). As MS progresses, clinical manifestations worsen. Promptly addressing MS-related spasticity symptoms/signs is crucial for optimising patient outcomes. However, in real-world clinical practise, detecting these symptoms is often delayed until they become severe or more evident, necessitating more aggressive pharmacological interventions such as higher doses or polypharmacy. In fact, the genesis of the spasticity symptom concept in MS stemmed from the observation of high medication burdens in patients as the disease advances, necessitating a reduction without compromising symptom control, alongside disease-modifying treatments (2). Therefore, the availability of a screening tool to detect SPS might help to standardise its evaluation and potentially contribute to reduce disease burden associated with polypharmacy.

The primary objective of this study is to develop a user-friendly tool that could help neurologists in promptly detecting the early detection of SPS in MS patients aiming for earlier intervention and potential improvement in patient outcomes.

## 2 Materials and methods

The methodological approach used to build an algorithm to identify SPS in MS patients was based in conjoint analysis principles (14). For this, a statistical orthogonal factorial design was employed to build and select 12 profiles of patients combining, at random, the presence or absence of the eight recognised principal signs/symptoms of SPS. This approach ensures that the effects of each factor (in our case, sign/symptom) can be estimated independently of the other factors, allowing researchers to assess the main effects of each factor as well as any interactions between. A definition of the signs and symptoms was agreed with the members of the scientific committee of the study (the authors of this paper) and included in the conjoint analysis exercise to ensure common interpretation (Table 1).

**Table 1:** Definitions of signs and symptoms of Spasticity-Plus Syndrome.

Sign/Symptom	Description
Spasticity	Muscular hypertonia characterised by velocity-dependent resistance to passive stretching, in a muscle or muscle group.
Spasms	Violent, sustained, and painful muscle contraction, in a muscle or muscle group.
Cramps	Spasmodic, involuntary, painful, and transient contractions, in a muscle or muscles.
Bladder dysfunction	Urinary urgency, incontinence, or tenesmus and/or nocturia.
Tremor	Abnormal involuntary movement, characterised by rhythmic oscillations, carried out by a part of the body or by the entire body, and around its axis of balance.
Fatigue	A feeling of exhaustion or decreased energy.
Sleep disorder	Nocturnal awakenings secondary to spasms or nocturia not associated with insomnia.
Pain	Unpleasant sensory and emotional experience like that associated with actual or potential tissue injury.

The 12 patient profiles were included in a survey questionnaire to be completed by a sample of Spanish MS experts. Each participant in this survey had to define, for each patient profile, whether it would be classified as presenting SPS or not (Table 2).

**Table 2:** Profiles of patients with possible Spasticity-Plus Syndrome included in the questionnaire.

Patient profile	Do you consider that this patient has *Spasticity-Plus Syndrome*? To answer, consider the definitions of the symptoms^*^
The patient has: Spasticity Spasms Fatigue and none of the other symptoms listed.	☐ Yes ☐ No
The patient has: Cramps Tremor Fatigue Sleep disorder and none of the other symptoms listed.	☐ Yes ☐ No
The patient has: Cramps Fatigue Pain and none of the other symptoms listed.	☐ Yes ☐ No
The patient has: Spasms Tremor Sleep disorder Pain and none of the other symptoms listed.	☐ Yes ☐ No
The patient has: Spasticity Spasms Cramps Sleep disorder and none of the other symptoms listed.	☐ Yes ☐ No
The patient has: Spasticity Spasms Cramps Bladder dysfunction Tremor Fatigue Sleep disorder Pain	☐ Yes ☐ No
The patient has: Bladder dysfunction Sleep disorder and none of the other symptoms listed.	☐ Yes ☐ No
The patient has: Spasticity Tremor Pain and none of the other symptoms listed.	☐ Yes ☐ No
The patient has: Spasms Cramps Bladder dysfunction Pain and none of the other symptoms listed.	☐ Yes ☐ No
The patient has: Spasticity Bladder dysfunction Fatigue Sleep disorder Pain and none of the other symptoms listed.	☐ Yes ☐ No
The patient has: Spasticity Cramps Bladder dysfunction Tremor and none of the other symptoms listed.	☐ Yes ☐ No
The patient has: Spasms Bladder dysfunction Tremor Fatigue and none of the other symptoms listed.	☐ Yes ☐ No

^*^See definitions of signs/symptoms in Table 1.

### 2.1 Survey sample calculation and participants

The size of the sample of participants was estimated based on the availability of experts in MS spasticity in Spain and considering the number of profiles to be evaluated. It was estimated that 80 neurologists would be needed to participate in the survey to evaluate patient profiles. For 12 profiles, a sample of 80 neurologists would provide up to 960 units of information for the conjoint analysis. This number was considered suitable for this intended analysis, since no exact rules for sample estimation are available in this setting.

Participants with no knowledge about SPS received a booklet including the main related publications so that they could familiarise themselves with SPS before responding in the conjoint exercise survey.

### 2.2 Statistical analysis

A descriptive analysis was performed to calculate the percentages of experts classifying each profile as SPS or not. Next, a logistic regression model was built to estimate the weight of each SPS sign/symptom in the decision to classify a profile as SPS or not. The constant of regression was fixed at 0, so that, in cases where none of the symptoms contributed at all to correctly classifying the profile as SPS, the model would give a probability of 0.50 (as with a random guess, since the dependent variable is dichotomous), and so that the regression coefficients would represent their relative comparative weight. The goodness-of-fit of the statistical model was estimated using the McFadden score and the area under the ROC curve (AUC).

The resulting coefficients of the model were used to classify the patient profiles as SPS or not, and Pearson’s and Spearman’s correlation coefficients were calculated to assess the similarity of the model’s classification distribution vs. the classification performed by the experts.

Optimal threshold was estimated using the ROC curve, and accuracy, sensitivity and specificity of the algorithm were estimated for the selected threshold.

## 3 Results

### 3.1 Description of participants

**Figure 1:**
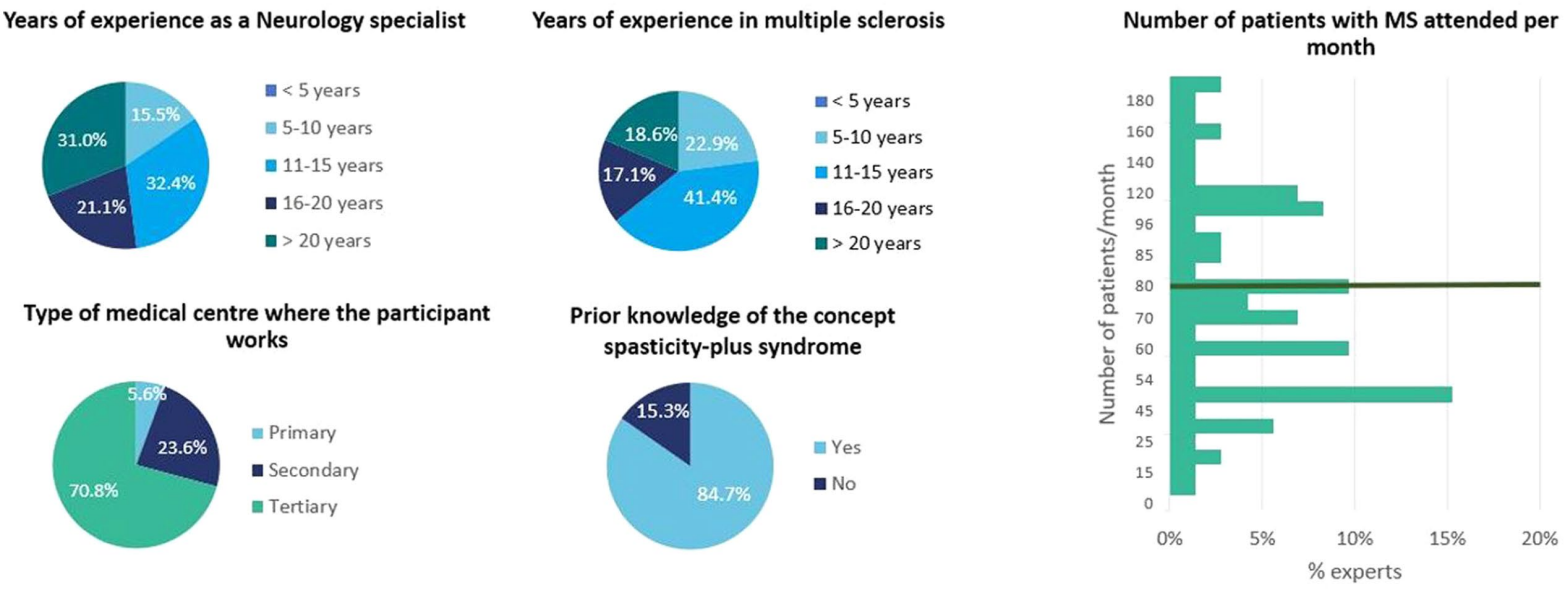
Characteristics of the participants in the conjoint exercise survey.

A total of 72 Spanish neurologists participated, responding in the conjoint exercise survey, which provided up to 864 units of information for the analysis. Geographical distribution of the experts was both wide-ranging and well-balanced, encompassing almost all the Spanish regions: Andalusia (*n* = 13), Aragon (*n* = 3), Asturias (*n* = 3), the Balearic Islands (*n* = 2), the Canary Islands (*n* = 1), Cantabria (*n* = 1), Castilla-Leon (*n* = 4), Castilla-La Mancha (*n* = 4), Catalonia (*n* = 9), Extremadura (*n* = 1), Galicia (*n* = 7), Madrid (*n* = 15), Murcia (*n* = 2), the Basque Country (*n* = 2), and Valencia (*n* = 5). 43% of participants were male, and all participants had extensive experience in the field of neurology, with a focus on multiple sclerosis. Most of the participants (70.8%) carry out their clinical practise mainly in tertiary care settings (hospitals). Most of the experts (84.7%) were already familiar with the SPS concept. The average number of patients attended per month by participants was 80.3 (Figure 1).

### 3.2 Evaluation of patient profiles by participants

**Figure 2:**
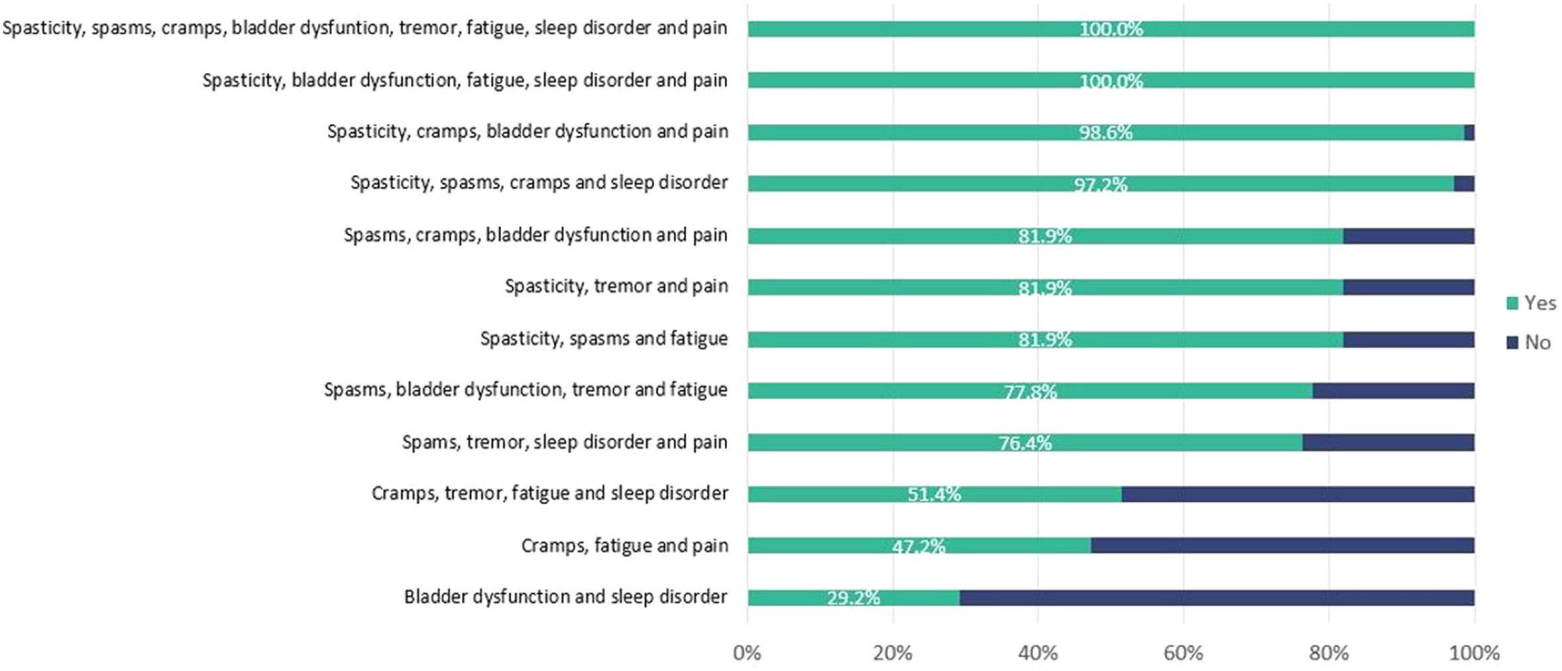
Responses of survey participants about the tested profiles of Spasticity-Plus Syndrome. Percentage of participants who answered ‘Yes’ (indicating that there is SPS) to each profile.

The survey results indicate that when all the eight symptoms are present, 100% of neurologists would consider that the patient has SPS. The same occurs when the profile includes spasticity, bladder dysfunction, fatigue, sleep disorder and pain. The degree of consensus amongst experts in the other profiles tested decreases depending on the combinations of signs/symptoms and particularly when spasticity or spasms are not present (Figure 2).

### 3.3 Contribution of each sign/symptom to the Spasticity-Plus Syndrome

The logistic regression results show the relative contribution of each individual sign/symptom to the probability of classifying a patient profile as SPS. Spasticity is the most influential sign, followed by spasms, tremor, cramps, and bladder dysfunction. In contrast, sleep disorder and fatigue had less decisive roles in identifying SPS. These findings are presented as coefficients of the logistic regression, odds ratios and 95% confidence intervals (CI), showing how the presence of specific signs/symptoms affects the likelihood of classification as SPS (Table 3).

**Table 3:** Results of the logistic regression model.

SPS sign/symptom	Logistic regression coefficient	95% CI of the coefficient	OR	95% CI of the OR
Spasticity	2.07^**^	1.65 to 2.52	7.92^**^	5.20–12.48
Spasms	0.77^**^	0.37 to 1.19	2.16^**^	1.45–3.28
Tremor	0.37^*^	−0.03 to 0.78	1.45^*^	0.97–2.19
Cramps	0.34^*^	−0.03 to 0.72	1.40^*^	0.97–2.05
Bladder dysfunction	0.3^*^	−0.05 to 0.64	1.35^*^	0.96–1.90
Pain	0.17	−0.19 to 0.52	1.19	0.83–1.68
Sleep disorder	−0.29	−0.63 to 0.04	0.75	0.53–1.04
Fatigue	−0.32	−0.69 to 0.04	0.73	0.50–1.05

CI, Confidence interval; OR, Odds ratio; and SPS, Spasticity-Plus Syndrome; ^*^p < 0.10; ^**^p < 0.001.

### 3.4 Adjustment of the model

The goodness-of-fit of the logistic regression model was assessed. The ROC curve indicates that the model significantly improves the likelihood of correctly classifying a profile as SPS vs. the random guess (50%), with an accuracy of 81%. A McFadden value of 0.21 indicates a good fit of the model. An AUC of 0.816 suggests effective profiling.

### 3.5 Evaluation vs. conjoint estimation of patient profiles

**Figure 3:**
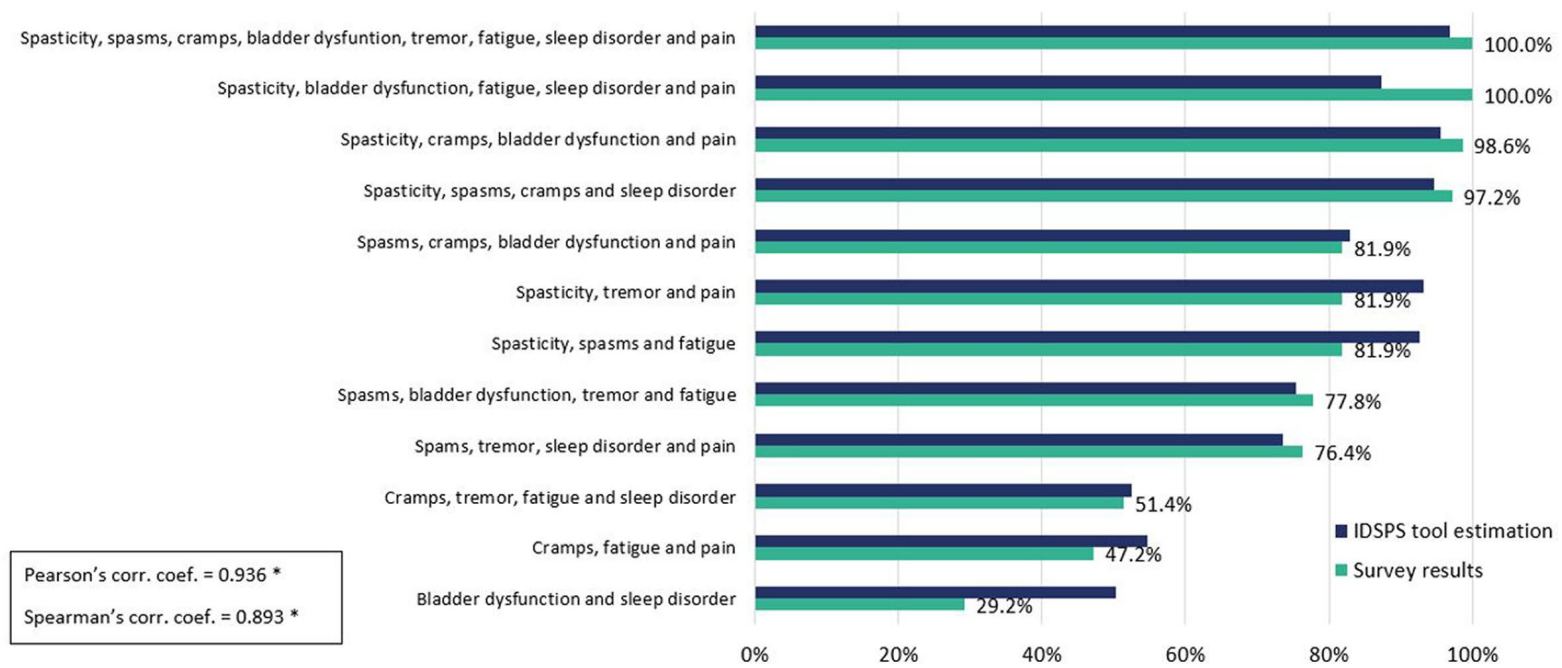
Survey results vs. model estimation of probability for each tested profile. Pearson and Spearman correlation coefficients. ^*^p < 0.001.

The adjustment of the model’s classification of patient profiles was also evaluated by means of a comparison between the model’s resulting probability of classifying each profile as SPS vs. the evaluation performed by the experts. The model’s data aligned well with the experts’ classifications, with strong Pearson’s (*r* = 0.936) and Spearman’s (*r* = 0.893) correlation coefficients between both distributions (Figure 3).

### 3.6 Sensitivity and specificity of the model

The model shows very adequate levels of sensitivity, specificity, and accuracy, considering its simplicity. For example, with a cut-off point of 0.74, the model shows accuracy of 0.76, sensitivity of 0.71, and specificity of 0.78. However, in this setting, authors consider that it is not advisable to define a cut-off point to decide whether a patient has SPS or not, but it is preferable to give the estimate of probability derived from the algorithm generated by the model, and with suggested indications based on ranges of probability which are explained below. Closed outcomes may not reflect the complexity of individual medical situation, therefore providing a probability complements the role of HCPs, who, with their clinical judgement, interpret the information provided by our (and other) tools.

### 3.7 Implementation of the algorithm for identification of the SPS—IDSPS tool

**Figure 4:**
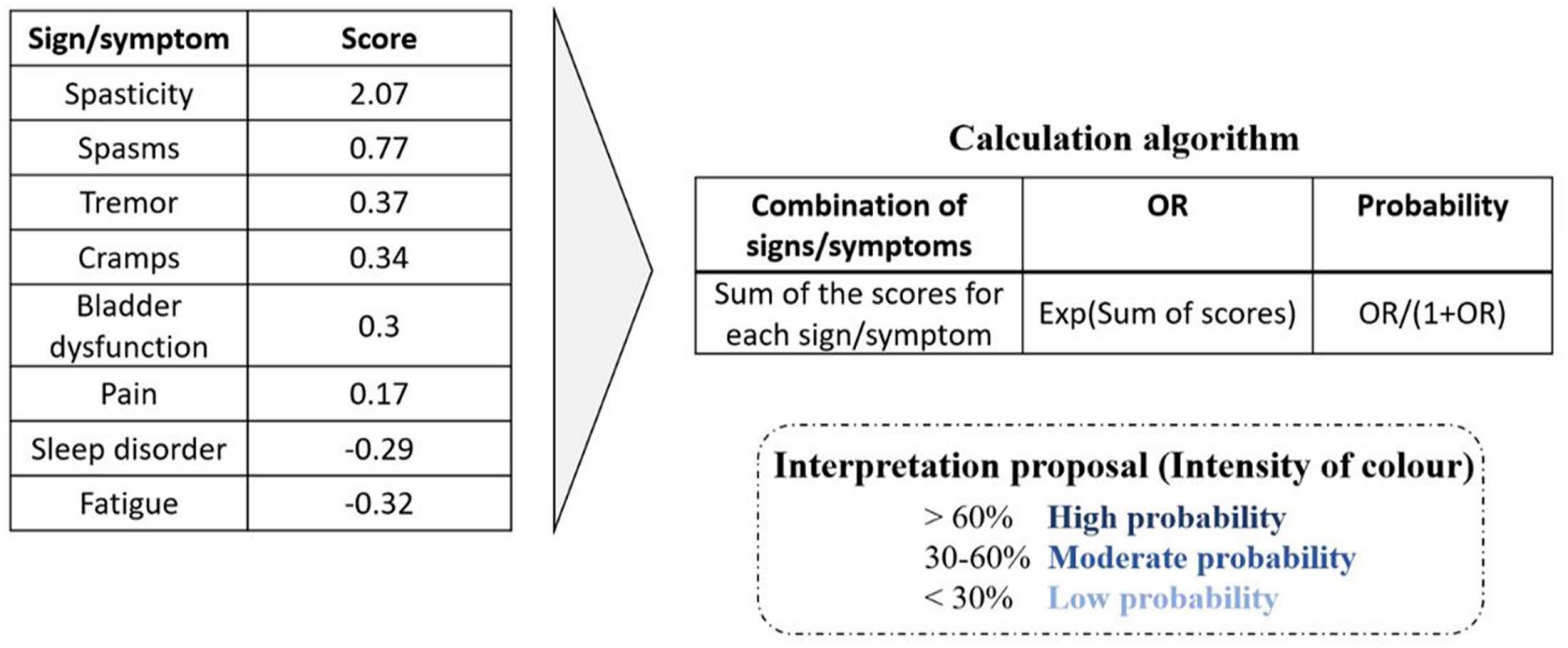
Calculation algorithm of the IDSPS tool.

**Figure 5:**
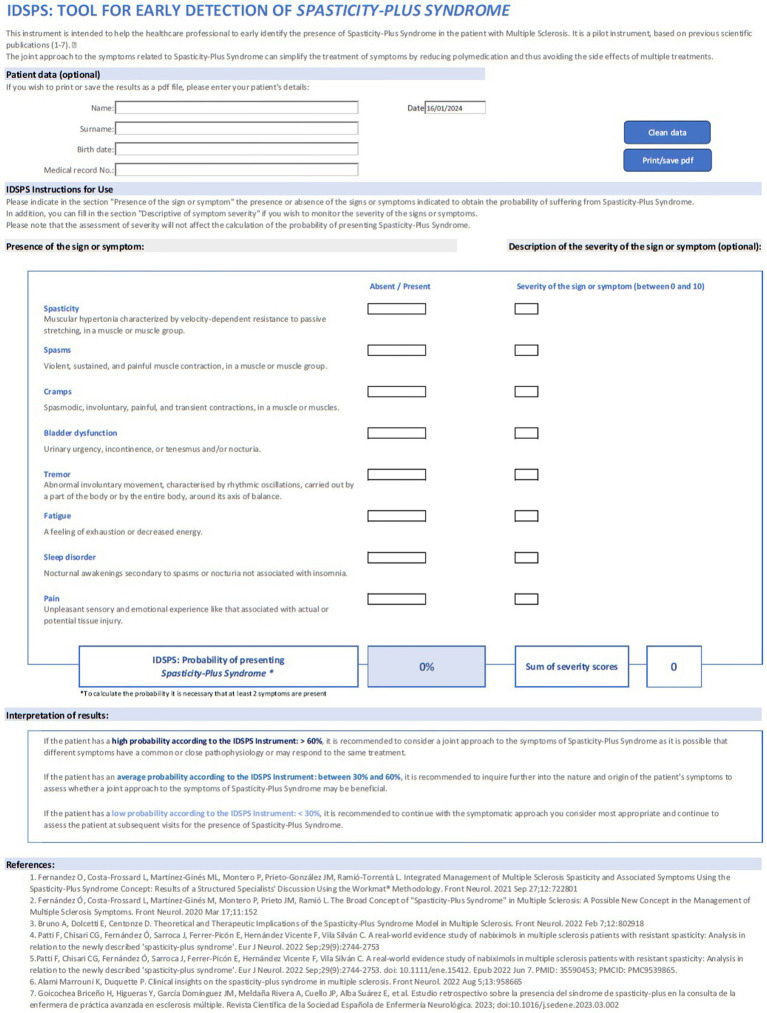
Image of the IDSPS tool.

Based on the results of this study, a prototype tool was developed to aid clinicians in the identification of SPS. The IDSPS tool includes the list of all eight symptoms included in SPS, and it is divided into two parts: an absent/present tick box and a severity scale. Once the absent/present tick box has been completed for all signs/symptoms, the tool will provide the probability that the patient would be identified as having SPS, by applying the calculation algorithm based on the logistic regression coefficients obtained in this model (Figure 4). The severity scale is incorporated to provide the clinician with additional information for a proper follow-up of the patient, but it does not influence the results from the algorithm. The algorithm classifies patient profiles into three suggested categories: high (> 60%), moderate (between 30 and 60%), and low (< 30%) probability of having SPS and provides with a recommendation in each case (Figure 5).

## 4 Discussion

This study describes the IDSPS tool that has been developed to help neurologists to detect SPS at an earlier stage in patients with MS. Diagnosis of SPS can simplify pharmaceutical treatment of symptoms in MS, which would help to avoid or reduce side effects of polypharmacy (2, 3, 10, 15, 16).

The broad concept of the existence of SPS has a double rationale. On the one hand, a possible relationship between several symptoms after increased muscle tone, and on the other hand, that the symptoms could be linked to the same area or in nearby areas of the brainstem (2, 15, 17). The improvement of such MS-symptoms with nabiximols has been observed in randomised clinical trials (5–9, 18) and in real-world studies (10–12, 19), where results have shown improvement not only in spasticity, but also in bladder dysfunction, sleep disorder and pain. The results of this conjoint analysis, based on the opinions of a group of selected neurologists, showed that the contributions of each individual symptom of the SPS to the probability of classifying a patient as SPS were spasticity as the most influential sign, followed by spams, tremor, cramps and bladder dysfunction coherent with the observation in clinical settings but less decisive role was observed for sleep and fatigue in our panel despite the available evidence supporting improvements in these symptoms (10–12, 19). These results based on the participant’s opinion reflect that, beyond the theoretical definition SPS, the different signs/symptoms do not contribute equally to SPS identification.

Polypharmacy is a common problem in MS patients and has been associated with lower quality of life (20–22), increased disability, comorbidities, cognitive deficits, fatigue, increased rates of hospitalisation and more frequent relapses (21). In the retrospective study by Goicochea Briceño et al. (16), in 85% of cases, the number of symptomatic treatments increased throughout the evolution of the disease, and the existence of polypharmacy in patients with SPS was confirmed. Management of multiple symptomatic therapies may be complex and require regular supervision and dose adjustments according to patient’s needs and treatment effectiveness and tolerability. The use of the IDSPS tool by neurologists would enable the detection of patients with SPS, and potentially a simplification of pharmacological treatment could be implemented. The adjustment of the therapeutic approach may help to the reduction of the side effects and improve patients’ quality of life.

In this study, the building phase of the IDSPS tool reveals an appropriate goodness of fit of the model and good levels of accuracy, sensitivity, and specificity of the model, supporting the validity of the tool.

The IDSPS tool is simple and easy to use and can therefore be used in clinical practise, even by nurses or other healthcare professionals; and it can be useful in making therapeutic decisions. The information provided by the IDSPS tool is clear for the neurologist, although the specialist is the one who will do the complete assessment to decide the best therapeutic approach in each case. The authors recommend using ranges of the shown probability to consider whether a patient should be managed as a case of SPS or not: < 30%—low probability; 30–60%—moderate probability; and > 60%—high probability of presenting SPS. Ranges are shown with different intensities of colour in the tool.

This study has some strengths and limitations. The notable strengths are: (1) Using a conjoint analysis approach to simulate patient profiles allows a better approach to clinical practise than simply assessing the weight of each sign or symptom separately; (2) The inclusion of definitions of the signs/symptoms in the survey and subsequent tool ensures consistency in the evaluation of the condition; and (3) The values of accuracy, sensitivity and specificity obtained by the algorithm are very reliable, considering the simplicity of the tool. The limitations of the study are: (1) The limited sample size did not allow to do a cross-validation of the model. It is therefore recommended to re-assess its validity after using the tool in clinical practise with a sufficient sample size; and (2) The conjoint analysis approach, although it mimics real patients by considering the pool of signs and symptoms simultaneously, is a theoretical exercise. Certainly, in the clinical practise setting, the clinician always has more information about the patient that can modify the decision to classify a patient as SPS or not.

## 5 Conclusion

This study provides an algorithm that could help neurologists in the consistent and efficient identification of MS patients with SPS. This can help to simplify the management of spasticity-related symptoms and to reduce the burden of side effects due to drug treatment. Clinical validation studies of the algorithm are needed to confirm the validity of the tool in clinical practise.

## Data availability statement

The raw data supporting the conclusions of this article will be made available by the authors under request, without undue reservation.

## Author contributions

ÓF: Writing – review & editing, Writing – original draft. LC-F: Writing – review & editing, Writing – original draft. MM: Writing – review & editing, Writing – original draft. PM: Writing – review & editing, Writing – original draft. JP: Writing – review & editing, Writing – original draft. LR-T: Writing – review & editing, Writing – original draft. YA: Writing – review & editing, Writing – original draft. AA: Writing – review & editing, Writing – original draft. EÁ: Writing – review & editing, Writing – original draft. AL-F: Writing – review & editing, Writing – original draft. LL: Writing – review & editing, Writing – original draft. AM: Writing – review & editing, Writing – original draft. EM: Writing – review & editing, Writing – original draft. PO-N: Writing – review & editing, Writing – original draft.
