# Supplementary material for: Practical tool to identify Spasticity-Plus Syndrome amongst patients with multiple sclerosis. Algorithm development based on a conjoint analysis
Source: Front Neurol. 2024 Apr 19;15:1371644. doi: 10.3389/fneur.2024.1371644 (PMC11066270; doi:10.3389/fneur.2024.1371644)
Supplement: Supplementary file 1 [file Presentation_1.PDF]

Supplementary material:

Practical information for using IDSPS tool to identify Spasticity-Plus Syndrome amongst patients with multiple sclerosis.

The IDSPS tool is an instrument developed to help clinicians to early identify Spasticity Plus Syndrome (SPS) in patients with MS. This tool contains the list of all eight symptoms included in SPS, and it is divided into two parts: an absent/present tick box and a severity scale.

Once the absent/present tick box has been completed for all signs/symptoms, the tool provides the probability that the patient could be identified as having SPS by applying the calculation algorithm based on the logistic regression coefficients described in the manuscript.

Use of IDSPS tool

The IDSPS tool is designed as a simple instrument that could be used in routine clinical practice to facilitate clinical assessment. After a patient exploration, neurologists may access to the IDSPS tool and complete the calculator fields.

Specialists should indicate in the section "Presence of the sign or symptom" the presence or absence of the signs or symptoms listed to obtain the probability of suffering from SPS.

In addition, if desired, the section "Descriptive of symptom severity" to monitor the severity of the signs or symptoms can also be filled. This assessment does not affect the calculation of the probability of presenting SPS.

If the results are wished to be printed or saved, the patient data can be entered.

Interpretation of the results

When the information required is completed the probability of presenting SPS is displayed as result. According to the algorithm the patients' profiles are classified into three suggested categories (also included as part of the tool instructions):

- Patient with a **high probability** of presenting SPS: >60%.
- Patient with a **moderate probability** of presenting SPS: between 30% and 60%.
- Patient with a **low probability** of presenting SPS: <30%.

Example 1:

A 40-year-old patient diagnosed of Multiple Sclerosis is explored and presents spasticity, cramps, bladder dysfunction, tremor and pain. When data is entered in the IDSPS, results revealed that the patient has a 96% probability of suffering from SPS.

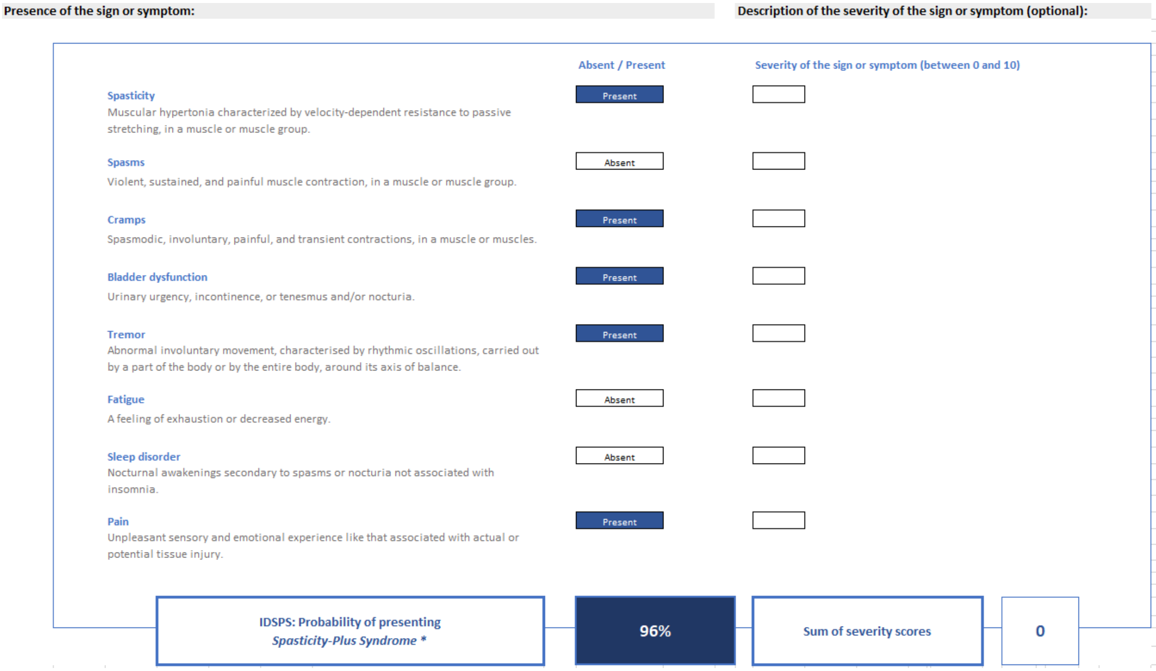

Figure 1. Sample screenshots from IDSPS tool. Example of completing the presence of the signs or symptoms options.

Example 2:

A 56-year-old patient diagnosed of Multiple Sclerosis is explored and presents cramps, fatigue, and sleep disorder. When data is entered in the IDSPS, result revealed that the patient has a 43% probability of suffering from SPS.

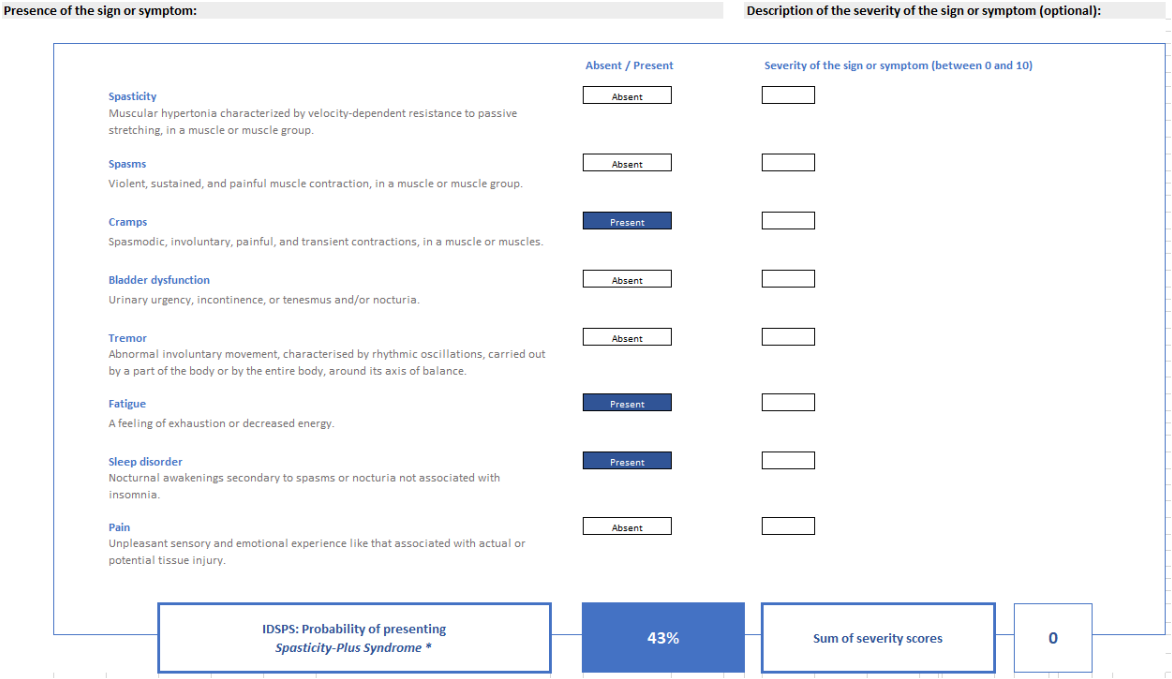

Figure 2. Sample screenshots from IDSPS tool. Example of completing the presence of the signs or symptoms options.
